# Cholinergic Potentiation and Audiovisual Repetition-Imitation Therapy Improve Speech Production and Communication Deficits in a Person with Crossed Aphasia by Inducing Structural Plasticity in White Matter Tracts

**DOI:** 10.3389/fnhum.2017.00304

**Published:** 2017-06-14

**Authors:** Marcelo L. Berthier, Irene De-Torres, José Paredes-Pacheco, Núria Roé-Vellvé, Karl Thurnhofer-Hemsi, María J. Torres-Prioris, Francisco Alfaro, Ignacio Moreno-Torres, Diana López-Barroso, Guadalupe Dávila

**Affiliations:** ^1^Cognitive Neurology and Aphasia Unit and Cathedra ARPA of Aphasia, Centro de Investigaciones Médico-Sanitarias, Instituto de Investigación Biomédica de Málaga, University of MalagaMalaga, Spain; ^2^Unit of Physical Medicine and Rehabilitation, Regional University Hospital, MalagaMalaga, Spain; ^3^Molecular Imaging Unit, Centro de Investigaciones Médico-Sanitarias, General Foundation of the University of MalagaMalaga, Spain; ^4^Department of Computer Languages and Computer Science, Superior Technical School of Engineering in Informatics, University of MalagaMalaga, Spain; ^5^Department of Psychobiology and Methodology of Behavioural Sciences, Faculty of Psychology, University of MalagaMalaga, Spain; ^6^Department of Spanish Language I, University of MalagaMalaga, Spain

**Keywords:** structural plasticity, neuroimaging, crossed aphasia, donepezil, aphasia therapy

## Abstract

Donepezil (DP), a cognitive-enhancing drug targeting the cholinergic system, combined with massed sentence repetition training augmented and speeded up recovery of speech production deficits in patients with chronic conduction aphasia and extensive left hemisphere infarctions (Berthier et al., 2014). Nevertheless, a still unsettled question is whether such improvements correlate with restorative structural changes in gray matter and white matter pathways mediating speech production. In the present study, we used pharmacological magnetic resonance imaging to study treatment-induced brain changes in gray matter and white matter tracts in a right-handed male with chronic conduction aphasia and a right subcortical lesion (crossed aphasia). A single-patient, open-label multiple-baseline design incorporating two different treatments and two post-treatment evaluations was used. The patient received an initial dose of DP (5 mg/day) which was maintained during 4 weeks and then titrated up to 10 mg/day and administered alone (without aphasia therapy) during 8 weeks (Endpoint 1). Thereafter, the drug was combined with an audiovisual repetition-imitation therapy (Look-Listen-Repeat, LLR) during 3 months (Endpoint 2). Language evaluations, diffusion weighted imaging (DWI), and voxel-based morphometry (VBM) were performed at baseline and at both endpoints in JAM and once in 21 healthy control males. Treatment with DP alone and combined with LLR therapy induced marked improvement in aphasia and communication deficits as well as in selected measures of connected speech production, and phrase repetition. The obtained gains in speech production remained well-above baseline scores even 4 months after ending combined therapy. Longitudinal DWI showed structural plasticity in the right frontal aslant tract and direct segment of the arcuate fasciculus with both interventions. VBM revealed no structural changes in other white matter tracts nor in cortical areas linked by these tracts. In conclusion, cholinergic potentiation alone and combined with a model-based aphasia therapy improved language deficits by promoting structural plastic changes in right white matter tracts.

## Introduction

The term *structural plasticity* refers to the brain’s ability to actually change its physical structure after repeated practice (Zatorre et al., 2012; Fridriksson and Smith, 2016). Very few studies have explored structural plasticity promoted by intensive therapy or non-invasive brain stimulation (NIBS) in aphasia (Allendorfer et al., 2012; Zipse et al., 2012; Wan et al., 2014). The available evidence suggests that location of structural plastic changes is not random as it probably depends upon the characteristics of the intervention [i.e., intensive Melodic Intonation Therapy in non-fluent aphasia targets the right arcuate fasciculus (AF)] (Schlaug et al., 2009; van Hees et al., 2014; Fridriksson and Smith, 2016). At present, there are no studies exploring whether structural plasticity can be enhanced combining a cognitive-enhancing drug with intensive therapy in chronic aphasia. Here, we report a significant improvement of aphasia severity, everyday communication and speech production (fluency and repetition) in a strongly right-handed male patient (JAM) with chronic conduction aphasia (CA) and a right subcortical hemorrhage (crossed aphasia) while he received the cholinergic agent donepezil (DP) and intensive audiovisual repetition-imitation therapy. Using pharmacological magnetic resonance imaging (phMRI) [diffusion weighted imaging (DWI) and voxel-based morphometry (VBM)] we documented plastic changes in both the right frontal aslant tract (FAT) and the direct segment of the arcuate fasciculus (DSAF).

The key role of cortical areas in speech production and communication deficits in aphasia is undisputed (Baldo et al., 2006; Borovsky et al., 2007). Nevertheless, the current notion is that spoken language and communication in normal conditions depend on large-scale networks that orchestrate the activity of specific brain regions via long-range white matter connections (Sassa et al., 2007; Willems and Varley, 2010; Simonyan et al., 2016; Halai et al., 2017). The impetus to examine structural plasticity in white matter tracts in JAM comes from findings of recent neuroimaging studies of white matter pathways underpinning speech production. A major contribution of two tracts [FAT and anterior segment of the arcuate fasciculus (ASAF)] to speech fluency together with other components of the speech production network has been demonstrated (Fridriksson et al., 2013; Basilakos et al., 2014). The FAT is a newly identified pathway in studies using post-mortem dissections (Vergani et al., 2014), direct electrostimulation (Vassal et al., 2014), and DWI (Klein et al., 2007; Ford et al., 2010; Catani and Thiebaut de Schotten, 2012; Catani et al., 2013, Kronfeld-Duenias et al., 2014; Broce et al., 2015). The FAT directly connects the supplementary motor area (SMA), pre-SMA and anterior cingulate areas with the pars opercularis of the inferior frontal gyrus (Catani et al., 2013; Vergani et al., 2014). Regarding the functions of cortical areas linked by the FAT, the pre-SMA is related to linguistic processing and cognitive control (Catani et al., 2013; Hertrich et al., 2016), whereas the SMA proper participates in speech motor control (initiation, coordination, and speech monitoring) (Laplane et al., 1977; Crosson et al., 2001; Alario et al., 2006; Hertrich et al., 2016). The pre-SMA and SMA participate on planning and motor initiation and interact with the executive motor cortex via the basal ganglia (motor loop) and thalamus (Bohland and Guenther, 2006; Bohland et al., 2010). Lesion mapping studies show that damage to medial frontal cortex (pre-SMA and SMA) interrupting (or not) the FAT correlates with speech arrest (Martino et al., 2012), reduced speech fluency (Catani et al., 2013; Basilakos et al., 2014; Kronfeld-Duenias et al., 2014), and impaired morphological derivation of verbs (Sierpowska et al., 2015). Another white matter tract implicated in speech fluency is the ASAF, which links the inferior parietal lobe with an inferior frontal region important for planning speech production (Marchina et al., 2011; Fridriksson et al., 2013; Basilakos et al., 2014; Pani et al., 2016). Although the classical arcuate fasciculus (AF), connecting the temporal with the frontal cortices has traditionally been related to verbal repetition (Geschwind, 1965), recent DWI-tractography studies have revealed a more complex picture of the AF connectivity (Catani et al., 2005). Thus, the AF may be divisible into three segments with anatomical termination differences, which support different functions. Precisely, verbal repetition has been linked with the activity of the long segment (the classical AF) and the posterior segment of the AF (Saur et al., 2008; Catani and Thiebaut de Schotten, 2012), whereas its anterior segment (ASAF) has been related to speech production and conversation (Catani et al., 2013). Despite that the ASAF overlaps with the FAT in the deep region beneath the Brodmann’s area 6, it has been suggested that damage to the ASAF and the FAT plays an independent yet synergistic deleterious effect on speech fluency in brain damaged subjects (Basilakos et al., 2014). The role of the uncinate fasciculus in speech fluency is more controversial (see Basilakos et al., 2014; Fridriksson and Smith, 2016; Hope et al., 2016).

An unsettled question is whether the structure of the white matter tracts mediating speech fluency can be successfully modeled with biological approaches (drugs, NIBS) and model-based aphasia therapies. Brain remodeling promoted by intensive aphasia therapies is increasingly being examined with neuroimaging. Intervention studies used repetition training in the presence of a picture (Heath et al., 2012) or embedded in Melodic Intonation Therapy (Sparks et al., 1974; Schlaug et al., 2009; Zipse et al., 2012) and Constraint-Induced Aphasia Therapy (Pulvermüller et al., 2001; Breier et al., 2011) with the aim of activating the remnants of left white matter pathways (i.e., AF) and/or to stimulate the compensatory activity of their homologs counterparts in the right hemisphere when the left ones are enduringly damaged. These studies showed improvements in picture naming (Heath et al., 2013; van Hees et al., 2014) and speech production (Schlaug et al., 2009; Breier et al., 2011; Zipse et al., 2012) and such benefits were attributed to therapy-promoted strengthening of auditory-motor assemblies or semantic-phonological connections in the right hemisphere (Heath et al., 2012; Zipse et al., 2012).

The effectiveness of rehabilitation to improve aphasia outcomes is often limited, particularly in patients with extensive damage to the language areas. Therefore, biological therapies (drugs and NIBS) are increasingly used to augment and accelerate the benefits provided by aphasia therapy. In previous studies, gains in speech production have been augmented combining model-based aphasia therapies and excitatory repetitive transcranial magnetic stimulation (rTMS) (Al-Janabi et al., 2014; see also Restle et al., 2012), excitatory (anodal) transcranial direct current stimulation (anodal-tDCS) (Vines et al., 2011) and cognitive-enhancing drugs (Berthier et al., 2014). Drug therapy plays an important role in the treatment of language deficits in chronic patients with post-aphasia (Berthier and Pulvermüller, 2011; Berthier et al., 2011; Llano and Small, 2016). Berthier et al. (2014) used massed sentence repetition therapy (40 h) to treat three patients with chronic post-stroke CA and large left hemisphere lesions who were receiving a cholinergic agent (DP). This combined intervention augmented and speeded up benefits in speech production deficits previously obtained in these patients with DP and distributed speech-language therapy (40 h) (Berthier et al., 2014). In recent years, however, speech pathologists recognize that auditory repetition practice alone is not enough to promote manifest benefits in everyday language activities and functional communication (Lee et al., 2010; Fridriksson et al., 2012, 2013). Therefore, training using repetition-imitation of audiovisual stimuli have been incorporated into the treatment of aphasia (Lee et al., 2010; Fridriksson et al., 2012, 2013; Heath et al., 2012, 2013). The rationale behind two such recently developed therapies, namely Intensive Mouth Imitation and Talking for Aphasia Therapeutic Effects (IMITATE) (Duncan and Small, 2016) and Speech Entrainment (Fridriksson et al., 2012, 2013), is using action observation and imitation of visual and auditory stimuli to enhance the activity of bilateral parietal-frontal pathways (audiovisual mirror neurons) (Mashal et al., 2012; Duncan and Small, 2016) and ventral language streams (Fridriksson et al., 2012, 2013). The idea behind these therapies was taken as a basis to develop our method called “Look, Listen and Repeat” (LLR) therapy^1^ to improve speech production deficits and communication in JAM.

## Materials and Methods

### Case Description

JAM was a 46-year-old right-handed, monolingual male with no history of neurological diseases, no family history of left-handedness, and normal developmental milestones (see further details in De-Torres et al., 2013). His personal history was remarkable for hypertension and type II diabetes. He suffered a right striatal-capsular hemorrhage associated with global aphasia, left hemianopia, and dense left hemiparesis with impaired sensation. According to the hospital report, as inpatient JAM suffered a single epileptic attack and at the time of discharge (15 days post-onset) he had fluent jargon aphasia and impaired comprehension. Reading and writing were also severely impaired. Four months post-onset (1 year before entering the drug trial), he showed depressive symptoms with a tendency to social withdrawal (Hamilton Depression Rating Scale score: 14 – Hamilton, 1960). A treatment with escitalopram (20 mg/day) was associated with an improvement of depressive symptoms in the next few months. The first formal evaluation of JAM in our Unit of Cognitive Neurology and Aphasia was performed 16 months after the hemorrhage. By that time, he showed a dense left hemiparesis (Fugl-Meyer Scale: left upper limb: 5/66; left lower limb: 7/34) (Fugl-Meyer et al., 1975), mild mobility problems (Rivermead Mobility Index: 13/15) (Collen et al., 1991) and dependency for some activities of daily living (bathing, dressing, feeding, and grooming) (Barthel Dependency Index: 50/100 – Mahoney and Barthel, 1965). On the Stroke Aphasia Quality of Life 39 (SAQoL-39) (Hilari et al., 2003; Lata-Caneda et al., 2009) he obtained an average overall score of 2.28 (physical 2.23, communication 3; psychosocial 1.8, and energy 2) indicating reduction of quality of life in all domains.

### Study Design

A single-patient, open-label multiple-baseline design incorporating two treatment and two post-treatment evaluations was used. An A-B-BC-D_1_-D_2_ design was used. After ensuring a stable baseline (A), the patient received DP (5 mg/day) during 4 week and then the dose was increased (10 mg/day) during 12 weeks without speech-language therapy in either phase (B). Thereafter, the patient continued with DP (10 mg/day) combined with LLR therapy (BC). After ending combined therapy, there were two washout periods of both DP and LLR therapy (D_1_-D_2_). Several comparisons between the different study phases were made including of A-B (week 0 vs. week 16), B-BC (week 16 vs. week 28), BC-D_1_ (week 28 vs. week 36), and BC-D_2_ (week 28 vs. week 44). Evaluations of language and communication deficits were performed at two baselines (week 0), endpoints B (week 16) and BC (week 28) and at washout periods (weeks 36 and 44). Other pharmacological treatments (escitalopram, losartan, sitagliptin/metformin, omeprazole, baclofen, and levetiracetam) were kept unchanged during the trial.

The study was performed according to the Declaration of Helsinki and the protocol was approved by the Local Community Ethics Committee for Clinical Trials and the Spanish Medical Agency. This single case study was conducted as part of Investigator-Initiated Research (IIT) funded by Pfizer/Eisai, Spain and it was designed, conducted, and controlled by the principal investigator (MLB). The study was registered with EudraCT number 2008-008481-12. The patient signed a written informed consent to participate in the study.

### Drug Treatment

The drug used was DP (orally disintegrating tablets of 5 and 10 mg). The dose used of DP (10 mg/day) represents the maximum dose used in well-designed studies of post-stroke aphasia (Berthier et al., 2006; Woodhead et al., 2017). Compliance was determined at every visit by tablet counts. DP tablets were provided by Pfizer/Eisai, Spain. The detection of potential adverse events was monitored during the trial.

### Aphasia Therapy

#### Stimuli Selection Procedure

All sentences included in the LLR therapy^2^ were composed of words of high-frequency, high-imageability, and predictability with an increasing length and grammatical difficulty. Individual words were selected from LEXESP (Léxico informatizado del español – Sebastián-Gallés et al., 2000). Sentences included words belonging to highly familiar semantic categories for both nouns (food, animals, places, transport, nature, household objects, everyday objects, nature, body parts, clothing, professions, ages, gender, and family) and adjectives (colors, sizes, appearances, and character). Three levels of difficulty were developed and there were several lists. The first level of difficulty contained three lists of 30 sentences construed with the following sentence structure: subject-verb (i.e., “The child runs”); verb-direct object (i.e., “Give me the bread”), and copulative sentence (i.e., “The child is nice”). The second level consists of four lists of 50 sentences each with sentences like: subject-verb-object (i.e., “The boy stood on a chair”), subject-verb-adjective (i.e., “The child runs nice”); and temporal/spatial complements: (i.e., “The child comes tomorrow”). It was planned a more complex third level that was not used with JAM because the second level was challenging enough to him and permitted a good working level. For the third level, a list of 25 complex sentences was construed. In the complex sentences the subordinate clause could be either adjectival (i.e., a relative clause as in “The boy who lives here is friendly”), nominal (i.e., “Angel believes that her mother will not come’) or temporal (i.e., “I’ll get it when I go home”).

#### Audiovisual Recordings

Five adult healthy subjects of both sexes (three females and two males) and varying ages (age range: 20–50 years) collaborated in recording the audiovisual stimuli. All five speakers were native speakers from Spanish. Only the speakers’ upper body and head were visible, and the hands were specifically excluded. The speakers were instructed to say the words and phrases as they would occur in everyday language. They were told to start and end each clip with the mouth closed, looking directly at the camera. For each stimulus, after the image of the speaker appeared in the laptop screen, there was a brief delay before the initiation of speech, followed by production of the phrase and finally a brief delay after the speaker had completed voicing of the phrase.

#### Treatment Procedure

In the baseline assessments, it was noted that JAM presented no difficulty in repeating single words and some non-words, showing variable difficulty with the repetition of two-word and three-word lists and sentences (see De-Torres et al., 2013). Therefore, the aphasia therapy program was tailored to treat the greater JAM’s difficulty: sentence repetition (see Salis et al., 2015; Eom and Sunga, 2016) with the aim of improving speech fluency (Kohn et al., 1990). JAM saw the face of a person in a videotape saying a sentence to the camera, and then he had a time of 5 s to repeat the prayer. The patient got both phonological and audio-visual input of oral and facial mimicry.

The lists were composed of 50 sentences each, except the first introductory three sentences (Level I) that were shorter (30 sentences). The average number of words per sentence in Level I was 4.46 and 6.10 in Level II. JAM was asked to repeat each list at least twice in the morning and twice in the afternoon 5 days a week for a period of 12 weeks (total training: ∼60 h). JAM was evaluated at home weekly in the execution of the therapy by one of us (ID-T). The lists were changed weekly when the objective (correct repetition ≥ 80% of stimuli) was achieved. Since immediate list repetition posed a sufficient degree of difficulty for JAM, training with lists using delayed repetition was not used.

### Baseline Testing

Language and communication were assessed twice before initiating the treatment. The first linguistic evaluation was performed in September 2011 (at the end of general cognitive testing) (see De-Torres et al., 2013), whereas the second linguistic evaluation was performed in April 2012. JAM patient did not receive any type of speech and language therapy between the first and second baseline evaluations.

### Outcome Measures

#### Aphasia Severity

The severity of aphasia was rated at baseline and at two different time points using the Western Aphasia Battery-Aphasia Quotient (WAB-AQ) (Kertesz, 1982). Two further evaluations were also carried out after ending both treatments. The WAB-AQ is a measure of aphasia severity, which is sensible enough to detect longitudinal changes after treatment of post-stroke aphasia with different cholinergic agents (Berthier et al., 2006; Chen et al., 2010; Hong et al., 2012; Yoon et al., 2015). Increases in the WAB-AQ scores ≥ 5 at the two endpoints (B and BC) and two washouts (D_1_ and D_2_) in comparison to baseline (A) were considered positive responses to the interventions (Cherney et al., 2010; Berthier et al., 2011).

#### Communication in Activities of Daily Living

Communication in activities of daily living was assessed with the Communicative Activity Log (CAL) (Pulvermüller and Berthier, 2008). The CAL was completed by the spouse of JAM in the presence of one member of the research team in order to clarify potential misunderstanding of questions’ content or scoring. The CAL is composed of 36 questions divided in two parts that address *quality of communication* (e.g., “How well would the patient verbally express criticisms or make complaints?”) and *amount of communication* (e.g., “How frequently would the patient verbally express criticisms or make complaints?”). The CAL’s quality of communication score is obtained by summing up scores for items 1–18. The amount of communication score is obtained by summing up scores over items 19–36. Scores range from 0 to 180 and high scores indicate better everyday communication. In previous intervention studies, the CAL has been found to be sensible enough to detect beneficial longitudinal changes (Berthier et al., 2006; Difrancesco et al., 2012; Kurland et al., 2012; Mohr et al., 2016).

#### Speech Production

To examine connected speech production, speech samples in one baseline, two treatment and two post-treatment phases were obtained from the Picnic Scene picture description of the WAB during a time limit of 5 min with the same methodology used in other patients with CA and treated with a similar therapy (Berthier et al., 2014). All descriptions were audiotaped and transcribed by one of us (MLB). There are no fully accepted rules for rating verbal production during picture description in aphasia. Although measures to rating spontaneous speech (fluency and information content) of the WAB have been used in previous studies (Basilakos et al., 2014) there is general agreement that these measures are to a certain extent unreliable (Gordon, 1998). In the present case, speech samples were analyzed using a more reliable methodology (Nicholas and Brookshire, 1993; Marchina et al., 2011; Zipse et al., 2012; Wang et al., 2013; Berthier et al., 2014). The following metrics were examined: number of words, number of words/minute (speech rate), correct information units (CIU), and percentage of CIUs. A CIU is defined as a non-redundant content words that convey correct information about the stimulus (Nicholas and Brookshire, 1993). To be classified as CIUs, words should be not only intelligible in context, but also accurate, relevant, and informative with respect to the stimulus (Nicholas and Brookshire, 1993). Meaningless utterances, perseverations, paraphasias, and other inappropriate information (exclamations) were counted as words, but not classified as CIUs. The percentage of correct information units (% CIU) was established using the following formula: number of CIUs/number of words × 100.

#### Repetition

##### Words

Repetition of words was evaluated with test 9 (Repetition: Imageability × Frequency) of the Psycholinguistic Assessments of Language Processing in Aphasia (PALPA) (Kay et al., 1992; Valle and Cuetos, 1995). This test contains 80 words presented in a mixed fashion. Words were grouped in four lists (20 items in each list) with variations in frequency and imageability. The lists contained high-frequency/high-imageability, high-frequency/low-imageability, low-frequency/high-imageability, and low-frequency/low-imageability words. These lists were matched for syllable length; items contained between one and four syllables. Although this PALPA subtest also contains 80 non-words, repetition of non-words was not evaluated in the trial.

##### Sentences

Repetition of sentences was tested with test 12 (Repetition: Sentences) of the PALPA battery (Kay et al., 1992; Valle and Cuetos, 1995). This task evaluates the ability to repeat auditory-presented sentences (*n* = 36) of different length (from 5 to 9 words). It is composed of reversible sentences (*n* = 20) and non-reversible (*n* = 16) sentences.

##### Idiomatic Phrases and Novel Phrases

Since the production of idiomatic expressions (also called formulaic language) primarily depends on the activity in right hemisphere neural networks (cf. Berthier et al., 2014; Stahl and Van Lancker Sidtis, 2015), a set of familiar idiomatic Spanish sentences (clichès) (*n* = 40) taken from the 150 Famous Clichès of Spanish Language (Junceda, 1981) was used in repetition. Moreover, previous studies on CA revealed a dissociation in the ability to repeat clichès as compared to novel sentences (McCarthy and Warrington, 1984). Therefore, a control a set of novel sentences (*n* = 40) matched with the idiomatic phrases was also tested for auditory repetition (Berthier et al., 2014). For example, for the idiomatic phrase: “Me lo dijo un pajarito” (“A little bird told me”) the novel control phrase: “Me lo dijo mi compadre” (“My friend told me”) was created.

### Reading and Writing

Oral reading was assessed with selected subtests of the PALPA battery (Kay et al., 1992; Valle and Cuetos, 1995). Reading of word and non-words was assessed with the Oral Reading: Letter Length (test 29) and Oral Reading: Non-words (test 36) subtests, respectively. Writing of words and non-words was tested with the Spelling to Dictation: Letter Length (Test 3) and Spelling to Dictation: Non-words (test 45) subtests, respectively. Only the scores of baseline assessment are shown.

## Pharmacological MRI

### Image Acquisition

The MRI studies were performed on a 3-T MRI scanner (Philips Gyroscan Intera, Best, The Netherlands) equipped with an eight-channel Philips SENSE head coil. Head movements were minimized using head pads and a forehead strap. A Short TI Inversion Recovery (STIR) was used to produce 24, 2.5 mm axial slices (interslice gap = 1 mm; TR = 4718 ms; TE = 80 ms; inversion time = 200 ms; 264 × 512 matrix; FOV = 230 mm; number of excitations = 2).

High-resolution T_1_-weighted structural images of the whole brain were also acquired for the patient JAM at three time points: Baseline, (week 0), DP (week 16), and DP-LLR therapy (week 28). T_1_-weighted scans were also obtained for 22 healthy control subjects, matched with JAM by sex (all controls were male) and age (mean age: 33.05 ± 10.03 years; range: 22–59 years). The acquisition sequence was three-dimensional magnetization prepared rapid acquisition gradient echo (3D MPRAGE), with the following parameters: acquisition matrix, 268/265; field of view, 224 mm; repetition time (TR), 9.2 ms; echo time (TE), 4.2 ms; flip angle,8°; turbo field echo (TFE) factor, 200; reconstruction voxel size, 0.68 mm × 0.68 mm × 0.8 mm. Two hundred ten contiguous slices were acquired, with 0 mm slice gap, the total acquisition time of the sequence was about 2 min and 50 s.

DTI data acquisition was performed for the patient at the three aforementioned time points, using multi-slice single-shot spin-echo echoplanar imaging (EPI) with specific parameters as follows: FOV = 224 mm × 224 mm × 120 mm, 2 mm thick slices with no gap, TE = 117 ms, TR = 12466 ms, reconstruction matrix = 128 voxels × 128 voxels, 32 diffusion directions with *b* = 3000 s/mm^2^, EPI echo train length: 59.

### Diffusion Weighted Imaging (DWI)

Diffusion weighted imaging data were analyzed using FSL, MRtrix3 v0.3.15^3^, NiBabel v2.1^4^ and Trackvis software packages. The data was denoized using MRtrix3. Motion and eddy current correction were performed using FSL. The estimated movements of the participants never exceeded 2 mm or 1.5° in any direction. A brain mask was generated using FSL. After that, the reconstruction and tracking of FAT and AF were carried out with MRtrix3 by combining the Constrained Spherical Deconvolution (CSD) reconstruction method (Tournier et al., 2007) with probabilistic streamlines tractography (Tournier et al., 2010, 2012). This significantly reduces the crossing fiber problem in diffusion images (Tournier et al., 2008). The main parameters used were: mask = whole brain mask, tracking algorithm = iFOD2, number of generated streamlines = 1.000.000. Also, in the case of FAT, the seed image was a 25 mm radius sphere in the pre-SMA and SMA, and in the case of AF was a 20 mm radius sphere in the inferior parietal lobule.

NiBabel was used to transform the obtained tractograms into a readable format for Trackvis, which allowed a flexible 3D visualization of the tracts. In particular, the output of tractography generation and the b0 image were used to generate the tract-files using tck2trk tool in NiBabel. Trackvis was used to visualize the tract-files. Two spheres in the posterior inferior frontal gyrus, pars opercularis (IFGOp) and pre-SMA/SMA were created to isolate the right and left aslant tracts (Catani and Thiebaut de Schotten, 2012). The three segments of the AF were dissected using three ROIs in the Broca’s, Wernicke’s, and Geschwind’s areas (Catani and Thiebaut de Schotten, 2012). Spurious fibers were removed from the tracks by using an additional avoidance ROI (logical NOT operation). The FAT and the AF were dissected in both cerebral hemispheres.

### White Matter Voxel-Based Morphometry

Voxel-based morphometry analysis was performed using statistical parametric mapping (SPM12)^5^, running on MATLAB R2013b (Mathworks Inc., Natick, MA, United States). All T1 structural images were AC-PC oriented. A lesion mask, drawn over the T1-weighted images of JAM for each time point, was applied to T1 images of the patient. Then, the T1-weighted images were segmented into gray matter, white matter, and cerebrospinal fluid tissue classes. They were normalized into the MNI space with modulation option and smoothed with an 8 mm FWHM kernel. The lesion masks were also normalized. A mask of the FAT and another one of the AF were generated using Trackvis v0.6.0.1^6^. In the case of FAT, the mask was based on the diffusion images (see next section) obtained at the third time point (DP-T). In the case of the AF, the left direct segment, the left posterior segment, the right posterior segment, and the right anterior segment were selected from the first time point (baseline) while the right direct segment and the left anterior segment were obtained from the second time point (DP alone). In all cases, the chosen tracts were those with larger volume and better definition. These masks were co-registered to each of the three T1-weighted images of the patient. To do so, the b0 image used to derive the mask was co-registered to each T1 scan using the FLIRT and FNIRT commands of FSL v5.0.9^7^. The obtained transformations were then applied to the masks. The co-registered masks were normalized into the MNI space by applying the forward deformation field of each T1-weighted image. The final masks were generated subtracting the lesion from the normalized FAT and AF masks. The white matter segment of each time point was then compared to the white matter segments of the controls in a VBM analysis. Only the areas of FAT and AF were studied, applying small volume correction (SVC) with the normalized masks for each time point. The applied contrast was Control > JAM. The contrast JAM > Control did not yield any significant results.

### Gray Matter Voxel-Based Morphometry

The grey matter segment at each time point (baseline, DP, and DP-LLR therapy) was also compared to the gray matter segments of the healthy controls in a VBM analysis. In particular, a SVC analysis was performed for each time point on cortical areas connected by the FAT and the AF, namely Broca’s area (Brodmann’s area [BA] 44, 45), pre-SMA and SMA (BA 6) and pars opercularis (BA 44) for the FAT, and, additionally, the inferior parietal cortex (BA 39 and 40) and temporal posterior (superior and middle) (BA 22, 41, 42, and 37) for the AF. The applied contrasts were Control > JAM and JAM > Control.

## Results

### Aphasia Severity and Communication Deficits

**FIGURE 1:**
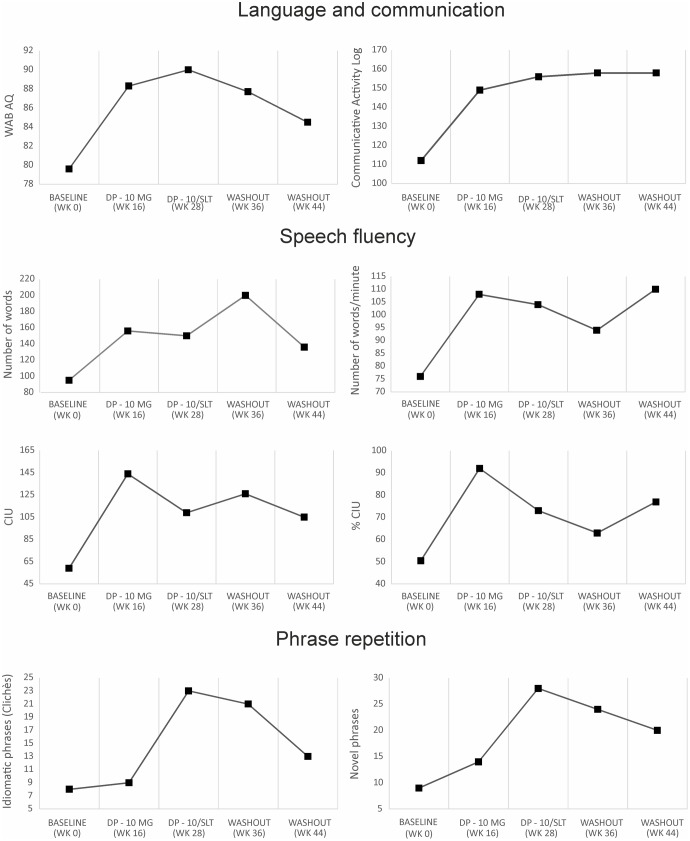
The graphs depict performance on language (WAB-AQ) and everyday communication (Communicative Activity Log), four measures of speech fluency, and repetition of idiomatic clichés and novel phrases at baseline, two endpoints, and two washout periods. The most impressive beneficial changes in language and communication and in measures of speech fluency (number of words, number of words per minute, correct information units [CIU] and % of CIU) were observed with donepezil (DP) alone (week 0 vs. week 16). As expected, the action of DP was enhanced on repetition of clichés and novel phrases during audiovisual repetition-imitation training (week 16 vs. week 28). WAB-AQ indicates Western Aphasia Battery. See further details in text.

Two baseline assessments revealed stable deficits in language (WAB-AQ, baseline 1: 78.8; baseline 2: 79.6) and everyday communication (CAL total, baseline 1: 113; baseline 2: 113) so that it seems that the benefits obtained in JAM were the direct effect of both treatments. A progressive improvement in both the WAB-AQ and CAL scores was observed at the two endpoints. Moreover, these benefits remained well-above baseline scores in the two washout evaluations performed several weeks after ending both interventions (**Table 1A** and **Figure 1**). On the WAB-AQ scores, treatment with DP alone (week 16) was associated with a gain of 8.7 points relative to baseline assessment (*p* = 0.008)^8^, an increment that allows classifying JAM as a responder to the drug (Cherney et al., 2010; Berthier et al., 2011). A further increment on the WAB-AQ (10.4 relative to baseline) when this dose of DP was combined with LLR therapy (week 28, *p* = 0.002). However, although scores on the WAB-AQ were higher with DP-LLR therapy than with DP alone, this difference did not reach statistical significance (*p* = 0.500). These gains remained stable in the first post-treatment evaluation (week 36, gain in AQ: 8.1, *p* = 0.008 compared to baseline) but not in the second post-treatment testing (week 44, gain in AQ: 4.9, *p* = 0.125 compared to baseline). Several verbal subtests of the WAB contributed to increase the AQ scores in the two endpoints, most notably (comprehension, repetition, and repetition)^9^.

**Table 1:** Performance of patient JAM on language, communication, speech fluency and repetition tasks at baseline, two endpoints and two washout evaluations.

Measures	Baseline (Wk 0)	DP-10 mg (Wk 16)	DP-10/SLT (Wk 28)	Washout-1 (Wk 36)	Washout-2 (Wk 44)
**(A) Language and communication**					
Western Aphasia Battery (WAB)					
Aphasia Quotient (max = 100)	79.6	88.3	90.0	87.7	84.5
Fluency (max = 10)	8	9	9	9	9
Comprehension (max = 10)	8.5	9.25	9.4	8.75	8.95
Repetition (max = 10)	6	7.4	7.8	7.7	8.4
Naming (max = 10)	8.9	9.5	9.8	9.4	6.4
Communicative Activity Log, total	112	149	156	158	158
Frequency	54	76	76	77	77
Quality	59	73	80	81	81
**(B) Speech fluency in connected speech**					
WAB – Picture description					
Number of elements described	14	18	20	18	18
Number of words	95	156	150	200	136
Number of words/minute	76	108	104	94	110
Time (seconds)	75	86	104	128	74
Correct information units (CIU)	59	144	109	126	105
% CIU	51	92	73	63	77
CIU/minute	38	100	76	59	85
**(C) Repetition of words and sentences**					
Word repetition (*n* = 80) (PALPA 9)	69	75	75	78	79
Sentences (PALPA 12)	8	11	12	10	11
Idiomatic sentences (clichès) (max = 40)	8	9	23	21	13
Novel sentences (max = 40)	9	14	28	24	20

The CAL’s total and its two subscales (quality and amount of communication) showed significant increases in comparison with baseline under treatment with DP alone (10 mg/day, week 16) (all *p* = 0.0001) and when DP was combined with LLR therapy (all *p* = 0.0001). Moreover, scores on CAL’s total and the quality of communication subscale improved more with combined DP-LLR therapy (week 28) than with DP alone (week 16) (both *p* = 0.016), but no changes were found in the amount of communication between these two endpoints (*p* = 0.100). The significant gains on CAL’s total score and on its subscales were maintained during the two washout evaluations (weeks 36 and 44) (both *p* = 0.0001).

### Connected Speech Production

All four parameters improved throughout the trial (**Table 1B** and **Figure 1**), yet the most noticeable gains were found under DP alone (weeks 0–16). There was a mild decrease in all these parameters with combined DP-LLR therapy (week 28) in comparison with DP alone (week 16). Improvements with both interventions remained well-above baseline scores in the two washout evaluations performed several weeks after ending the trial.

### Repetition

Repetition of words (PALPA 9) showed significant improvements in the two endpoints (weeks 16 and 28) in comparison with baseline assessment (both *p* = 0.031), but there were no differences between them (*p* = 0.100) (**Table 1C** and **Figure 1**). Improvements were maintained in both post-treatment evaluations (weeks 36 and 44) (both *p* < 0.005). No significant benefits were found in Sentence Repetition (PALPA 12) with either intervention. Treatment with DP alone (week 16) failed to improve repetition of idiomatic (clichès) phrases (*p* = 0.100), but there was a strong trend for improvement in repetition of novel phrases (*p* = 0.063). The combined intervention with DP-LLR therapy (week 28) significantly improved JAM’s performance on repeating idiomatic clichès and novel phrases (both *p* = 0.0001) and this intervention was significantly better than the treatment with DP alone (week 28 vs. week 16; both tasks *p* = 0.0001). In addition, these gains remained stable in both post-treatment evaluations for novel sentence repetition (week 36: *p* = 0.0001; week 44: *p* = 0.001) and only at the first post-treatment evaluation for idiomatic clichès repetition (week 36, *p* = 0.0001) although there was a trend for improvement in this task at the second post-treatment evaluation (week 44, *p* = 0.063).

### Reading and Writing

Scores on reading and writing are only shown for baseline assessment. Reading testing revealed that JAM showed a profile bordering between phonological and deep dyslexia (Oral Reading: Letter Length: 24/24; Oral Reading: Non-words: 13/24). In fact, JAM could read more than 50% of non-words correctly and he produced only few semantic errors. Writing showed that JAM the typical profile of deep dysgraphia (Spelling to Dictation: Letter Length: 15/24; Spelling to Dictation: Non-words: 0/24) with a profound deficit on non-word writing and several semantic errors.

### Sensorimotor Deficits

No changes were observed in the severity of left sensorimotor deficits with different therapeutic interventions (physiotherapy, DP alone and combined with aphasia therapy).

## Neuroimaging Findings

### Lesion Location and Volume

**FIGURE 2:**
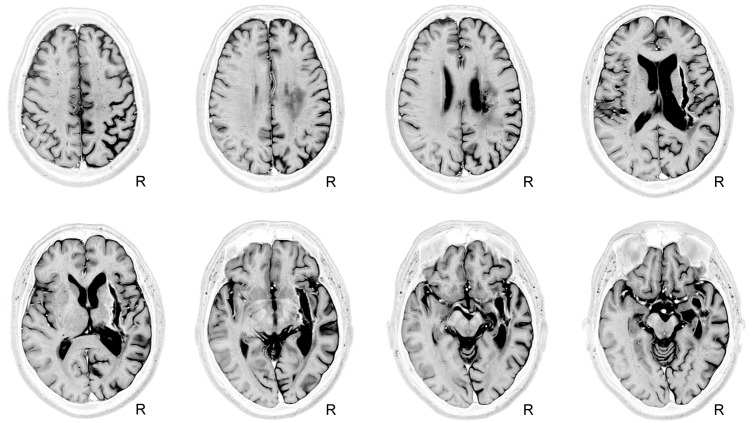
Depiction of an old right subcortical hemorrhage on a Short-TI Inversion Recovery (STIR) MRI sequence. Axial views of MRI-STIR images are shown in native space. The MRI shows an extensive lesion with a semilunar configuration involving the right striatum-capsular region extending into the surrounding white matter. Mild post-stroke right temporal-parietal cortical atrophy is evident. See text for further details. The neurological convention is used. R, right.

**FIGURE 3:**
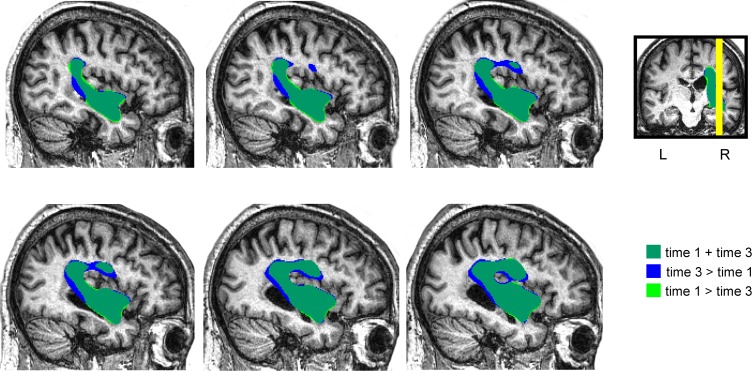
Depiction of the patient’s lesion mask in the time point 1 (BL) and in the time point 3 (DP + T) over the patient’s normalized sagittal T_1_-weighted image. The lesion mask drawn of the initial MRI (16 months post-stroke onset) was larger in the third MRI (28 weeks after study entry) due to expansion of its subinsular component. The observed lesion expansion probably resulted from retraction of cortical tissues due to focal temporal-parietal post-stroke atrophy (see **Figure 2**).

The structural MRI showed a large deep lesion involving the putamen, part of the external pallidum, and anterior limb, genu, and posterior limbs of the internal capsule of the right hemisphere (**Figure 2**). The lesion extended superiorly to the periventricular white matter (corona radiata). There also was tissue damage in the white matter surrounding the hippocampus and the middle temporal gyrus with posterior extension to the auditory and optic radiations in the temporal lobe. The right posterior ventral and dorsal insular cortices and the periventricular white matter deep to the supramarginal gyrus were also involved. It was noteworthy that although the initial MRI scan was obtained in the chronic period (16 months post-stroke onset), the volume of the lesion expanded in the third MRI (week 28 after study entry) compared to the first one due to enlargement of its more superficial components at the level of the insular cortex (**Figure 3**). The observed lesion expansion probably resulted from retraction of cortical tissue in the mid-temporal region due to focal temporal-parietal post-stroke atrophy (see **Figure 2**).

### Diffusion Weighted Imaging: Tractography

**FIGURE 4:**
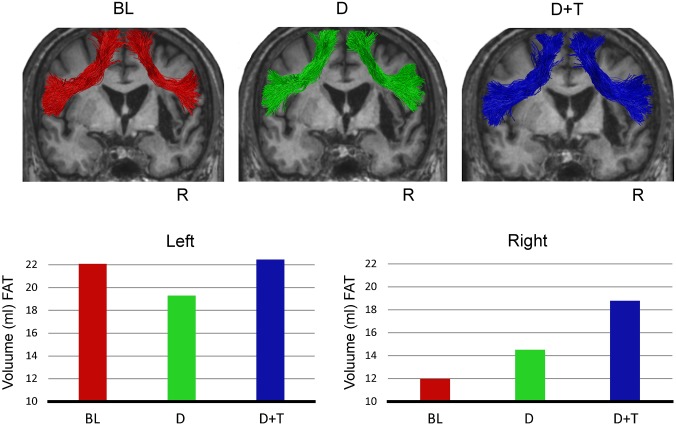
Tractography reconstruction of the left and right frontal aslant tracts (FAT) on the coronal plane in the three different time points. White matter microstructural changes are observed in the FAT in the baseline (BL), after drug treatment with donepezil (D) alone and after combined Donepezil and therapy (D + T). At the top, the FAT is showed bilaterally over imposed on the T_1_-weighted patient’s image in native space. At the bottom, the FAT volume is plotted for each hemisphere. Note that the volume pattern of the left FAT is more stable than the volume of the right FAT which increases progressively across the study phases. Neurological convention is used. R, right.

*In vivo* dissection using two ROIs approach of the FAT (Catani et al., 2013) revealed that this pathway was preserved in the right hemisphere in spite of its proximity with the lesion (**Figure 4**). Volume was measured along the reconstructed FAT streamlines independently for right and left hemispheres for each of the three time points. Volume measures in the initial evaluation (Baseline: left FAT: 22.08 ml; right FAT: 11.97 ml) suggested an asymmetrical pattern of distribution which became more symmetrical across the next evaluations (DP: left FAT: 19.29 ml; right FAT: 14.51 ml; DP-LLR therapy: left FAT: 22.45 ml; right FAT: 18.77 ml). To confirm this finding, a lateralization index (LI) was calculated as follow: (Right volume – Left volume)/(Right volume + Left volume). The LI has previously been used to assess microstructural differences in white matter pathways between the cerebral hemispheres (Catani et al., 2007; López-Barroso et al., 2013). The LI ranges between -1 and +1, where negative values represent left lateralization, values around zero represent symmetrical distribution, and positive values a right lateralization. The patient’s FAT showed a LI = -0.29 in the baseline phase; LI = -0.14 in DP phase; and LI = -0.08 in DP-LLR therapy phase. Thus, the FAT showed a more symmetrical pattern of distribution after the combined DP-LLR therapy treatment, suggesting that the structural reorganization of this pathway was related to the intervention and the associated improvements in fluency.

Virtual dissection of the AF was performed separately for the anterior, the posterior, and the direct segments using a 2 ROIs approach in both hemispheres. The three segments of the AF were reconstructed bilaterally. Volume measures for the three segments along the three time points showed different patterns of symmetry (Baseline: left anterior segment: 16.45 ml; left posterior segment: 11.5 ml; left direct segment: 42.1 ml; right anterior segment: 17.25 ml; right posterior segment: 6.93 ml; right direct segment: 13.37 ml; DP: left anterior segment: 18.8 ml; left posterior segment: 9.21 ml; left direct segment: 39.86 ml; right anterior segment: 13.53 ml; right posterior segment: 2.94 ml; right direct segment: 16.59 ml; DP-LLR therapy: left anterior segment: 16.6 ml; left posterior segment: 8.89 ml; left direct segment: 31.2 ml; right anterior segment: 14.08 ml; right posterior segment: 4.21 ml; right direct segment: 17.48 ml). The LI revealed that the direct segment was more left lateralized at the baseline evaluation (LI = -0.51) and became more symmetrical (DP, LI = -0.41; DP-LLR therapy LI = -0.28).

### White Matter Voxel-Based Morphometry

**FIGURE 5:**
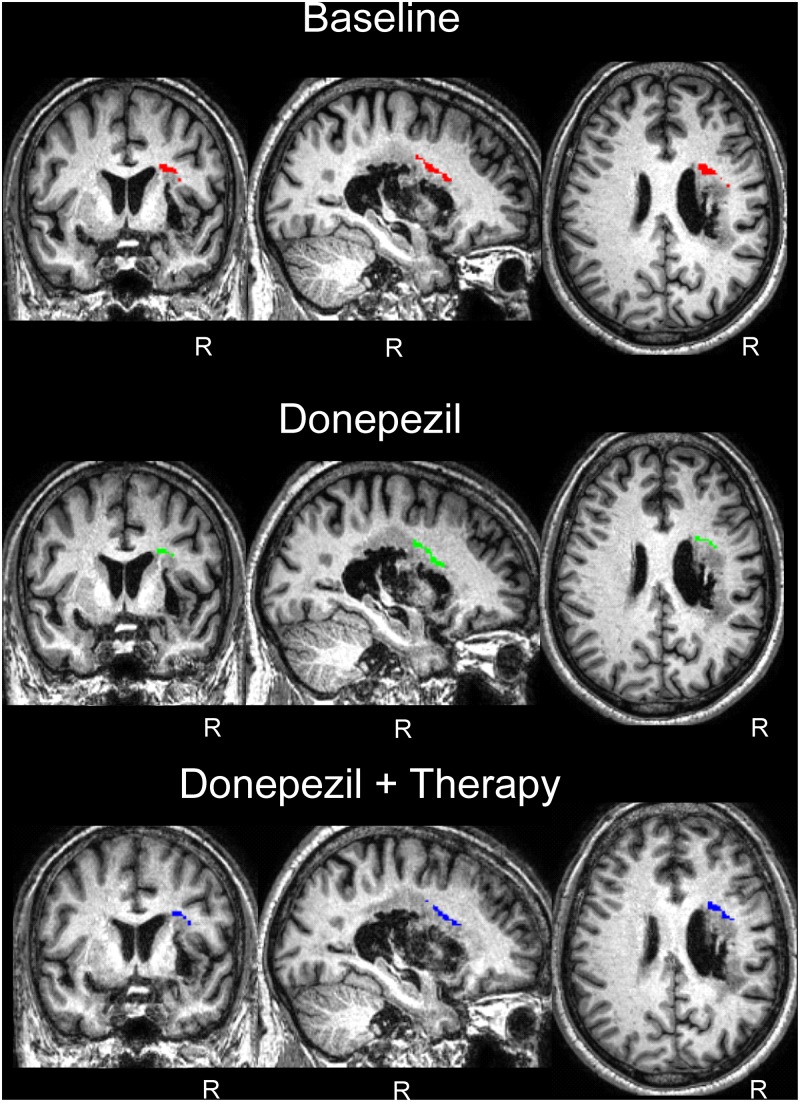
White matter voxel-based morphometry (VBM) results in the FAT region of interest. Control group > Patient JAM: compared to patient JAM, the control group presented greater white matter volume in the region corresponding to the FAT in the right hemisphere (see **Table 2** and “Results”). This difference decreases after the DP phase, and after the DP-LLR therapy phase. No differences were found in the left hemisphere. Neurological convention is used. R, right.

**FIGURE 6:**
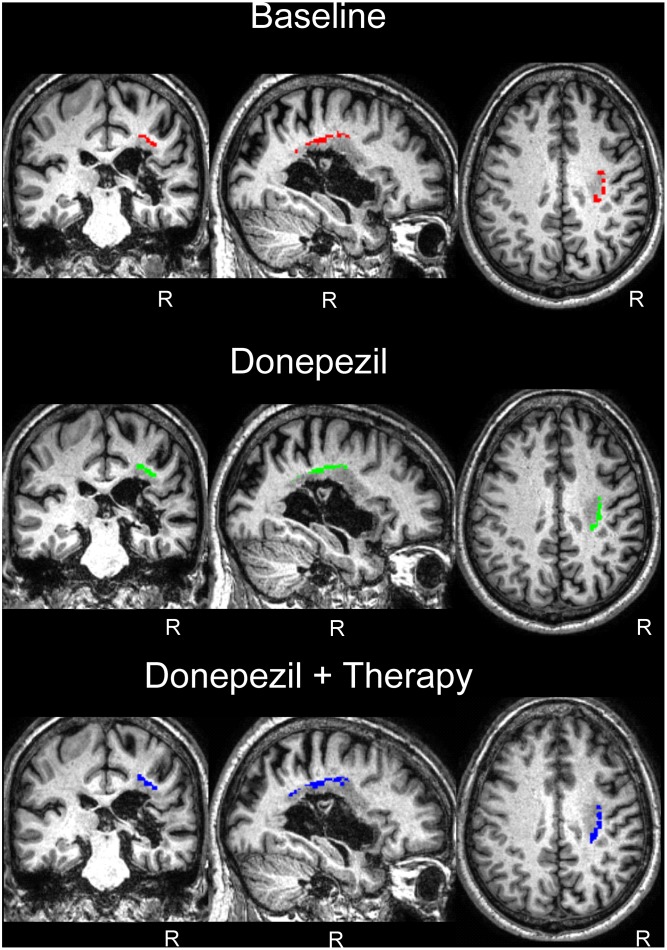
White matter VBM results in the arcuate fasciculus region of interest. Control group > Patient: compared to patient JAM, control group presented greater white matter volume in the region corresponding to the arcuate fasciculus in the right hemisphere (see **Table 3** and “Results”). No differences were found in the left hemisphere. Neurological convention is used. R, right.

The white matter volume of each time point was compared to the white matter of the controls in a VBM analysis for regions of interest comprising the regions of the FAT and the AF. For the FAT, the applied contrast Control > JAM revealed different significant clusters in the white matter which correspond with the FAT in the right hemisphere, showing that the volume in these regions was lower in JAM compared to controls (**Figure 5**). The total number of voxels comprised in the clusters decreased over the different evaluations (**Table 2**) indicating that the local volume of the ROI in JAM was more similar to the healthy brain after the DP and DP-LLR therapy phases. These results converge with the tractography volume analysis (see previous section). For the AF, the contrast Control > JAM also revealed different clusters showing lower volume lower in JAM compared to controls. However, contrary to the pattern found in the tractography analysis, here the cluster was substantially bigger across the different evaluations (**Table 3** and **Figure 6**).

**Table 2:** White matter voxel-based morphometry of the FAT in patient JAM at baseline and two endpoints compared to healthy control subjects.

		pFWE-corr	*T*	Peak coordinates (MNI)			kE
**FAT**	Baseline	0.007	8.5	24	3	26	177
		0.018	7.89	36	-3	27	
		0.042	7.37	28	-4	33	
	Donepezil	0.005	8.74	28	-3	30	166
		0.007	8.54	26	6	21	
		0.024	7.73	36	-3	27	
	Donepezil + Therapy	0.006	8.66	32	2	26	160
		0.024	7.73	28	9	21	
		0.083	6.94	28	-3	33	
		0.024	7.73	28	9	21	

*pFWE-corr indicates p Family Wise Error-corrected; T, *t*-value; MNI, Montreal Neurological Institute; kE, cluster extent; FAT, Frontal Aslant Tract*.

**Table 3:** White matter voxel-based morphometry of the Arcuate Fasciculus in patient JAM at baseline and two endpoints compared to healthy control subjects.

		pFWE-corr	*T*	Peak coordinates (MNI)			kE	Ke in AS/DS/PS
**AF**	Baseline	0.002	9.45	32	-24	33	212	206/189/0
		0.010	8.28	34	-14	33		
		0.021	7.81	28	-36	32		
		0.029	7.60	32	-15	38	38	36/27/0
		0.935	4.91	32	-6	36		
		0.065	7.09	36	-44	27	19	8/19/0
	Donepezil	0.003	9.15	32	-26	33	297	289/252/0
		0.020	7.85	34	-12	33		
		0.025	7.69	27	-34	33		
	Donepezil + Therapy	0.000	10.54	32	-12	33	306	297/265/0
		0.002	9.49	32	-30	32		
		0.003	9.05	28	-24	38		
		0.146	6.58	32	-46	27	16	8/15/0
		0.197	6.38	38	-42	30	13	12/13/0

*pFWE-corr indicates p Family Wise Error-corrected; T, *t*-value; MNI, Montreal Neurological Institute; kE, cluster extent, AF, Arcuate Fasciculus*.

### Gray Matter Voxel-Based Morphometry

The gray matter SVC comparison between JAM and the healthy control group did not yield any significant results for any of the studied areas at any of the time points.

## Discussion

In the present study, treatment with DP alone and combined with LLR therapy improved aphasia severity, communication, and measures of speech fluency and phrase repetition in JAM, a patient with crossed CA aphasia. The improvement of a long-lasting language and communication deficits in JAM may be attributed to regional structural neuroplastic changes in the right FAT and DSAF. However, no beneficial changes were observed after DP and physiotherapy in the left sensorimotor deficits, a finding that was at variance with data reported in a very similar patient with crossed CA and a right subcortical infarct who recovered left foot mobility (Berthier et al., 2003). JAM had phonological-deep dyslexia and deep dysgraphia (Crisp and Lambon Ralph, 2006). Description of the pattern and longitudinal evolution of these deficits is beyond the scope of this report and will be reported elsewhere.

Before advancing further in the interpretation of our results, data from JAM should be interpreted with caution because he had atypical brain-language organization (see De-Torres et al., 2013). JAM had both an atypical lateralization of language in the right hemisphere and a rare form of crossed aphasia (subcortical CA) (Alexander et al., 1989; Jung et al., 2010). These atypicalities prevent extrapolating the results obtained in JAM to other aphasic patients with typical lateralization and intrahemispheric organization of language functions in the left hemisphere. Moreover, since we studied a single case the causality of neuroplastic changes found with DWI and VBM in the right FAT and DSAF remains unclear. Finally, the open-label, uncontrolled design of our study is another limitation.

Despite these limitations, the present case study introduces new evidence to the few studies reporting the use of novel therapeutic interventions to treat crossed aphasia (Raymer et al., 2001; Jung et al., 2010; Lu et al., 2014). Until now, only three patients with chronic crossed aphasia have been treated with either drugs or NIBS. Raymer et al. (2001) treated a patient with crossed transcortical motor aphasia with the dopamine agonist bromocriptine. Dopaminergic stimulation produced long-lasting benefits in verbal fluency (words/minute in discourse), even after drug withdrawal, with little improvement in emotional prosody and gestural tasks. Language deficits in two patients with crossed aphasia were treated with inhibitory (1 Hz) rTMS over different cortical areas of the left hemisphere. One patient had chronic CA secondary to a right basal ganglia hemorrhage (Jung et al., 2010). After a short trial of rTMS over the left parietal lobe, improvements in language were restricted to the naming subtest of the WAB (pre-rTMS: 54/100; post-rTMS: 64/100) with no changes in fluency (pre-rTMS: 11/20; post-rTMS: 11.5/20). Post-treatment fMRI showed significant activations in the right inferior frontal gyrus, posterior temporal gyrus, and parietal lobe for both the noun generation and sentence completion paradigms (Jung et al., 2010). The other patient had an early chronic Broca’s aphasia associated to a large right hemisphere cortical-subcortical infarction, which disrupted functional and structural connectivity of right hemisphere networks (Lu et al., 2014). Stimulation over the posterior left temporal cortex improved auditory comprehension, whereas stimulating the left frontal cortex enhanced expression (naming). Gains in the WAB-AQ scores continued to increase during 6 months after ending brain stimulation (Lu et al., 2014).

### Language and Communication

Treatment with DP alone and combined with LLR therapy in JAM improved aphasia severity (WAB-AQ) and deficits in everyday communication (CAL). This parallel improvement was not unexpected. In a previous study, we demonstrated that a combined intervention with DP and conventional speech-language therapy in patients with chronic post-stroke aphasia and left hemisphere lesions significantly improved both domains (Berthier et al., 2006). Speech fluency, auditory comprehension and repetition subtests of the WAB showed improvement in JAM, which is agreement with the results of previous studies showing that gains after training repetition alone (for recent reviews see Salis et al., 2015; Eom and Sunga, 2016) and combined with drugs (Berthier et al., 2014) can generalize to other language domains. The improvement in everyday communication in JAM is relevant because deficits in everyday communication are strongly related to overall aphasia severity (Fucetola et al., 2006; Mazaux et al., 2013) and because recovery of spoken language in many aphasic patients rarely “scale up” from fragmented and paraphasic emissions to more cohesive and efficient everyday communication. It is important to emphasize that both interventions (DP and DP-LLR therapy) improved quality but not amount of everyday communication. This dissociation was expected because at baseline assessment JAM had fluent spontaneous speech (see next section), but the content of his emissions was contaminated by hesitation, some phonemic paraphasias, and occasional self-corrections (De-Torres et al., 2013). Therefore, there was more room for improvement in quality than for amount of communication.

Although language and communication are inherently linked to convey a coordinate message during social interaction, these functions may be dissociable by virtue of depending on the activity of different cortical areas (Sassa et al., 2007; Willems and Varley, 2010; Catani and Bambini, 2014). Spoken production depends on the activity of left perisylvian areas, whereas the intention to communicate relies on the activity of the left medial frontal cortex (pre-SMA, SMA, and anterior cingulate gyrus). Since these distant cortical areas are connected via the FAT (Catani et al., 2013; Hartwigsen et al., 2013), it is possible that modeling the right FAT with DP and DP-LLR therapy could have heightened the propagation of neural impulses between the medial frontal cortex important for modulating communicative intentions and the inferior frontal gyrus mediating spoken production. Cortical areas connected by the FAT showed no changes with either intervention.

### Speech Fluency and the Frontal Aslant Tract

Treatment with DP alone in JAM improved the scores on the experimental measures of speech fluency (efficiency and speech rate). These gains slightly decreased with combined DP-LLR therapy and after ending both interventions, thus indicating that the drug alone provided the most noticeable effects. JAM had a fluent aphasia obtaining a high baseline score on speech fluency of the WAB (8/10) and this score showed no significant improvement (9/10) throughout the trial. Although stability on this 10-point WAB scale may reflect a ceiling effect, it is also possible that this metric has failed to capture changes (Gordon, 1998) promoted by the treatments.

Previous cross-sectional neuroimaging studies in patients with post-stroke aphasia (Marchina et al., 2011; Fridriksson et al., 2013; Wang et al., 2013; Basilakos et al., 2014) and primary progressive aphasia (Catani et al., 2013) as well as computational implementations (Roelofs, 2014) collectively suggest that the FAT and the ASAF play a synergistic role to support speech fluency during communication. In our longitudinal study of patient JAM, we found a coincident increase of volume in the right FAT with reduction of its volumetric difference relative to healthy controls with DP alone and DP-LLR therapy. By contrast, the right ASAF instead showed a steady decrement in volume and increased volumetric difference when compared with healthy controls throughout the trial. Thus, both interventions induced circumscribed structural plasticity in one white matter tract coupled with shrinkage of the other. These divergent changes are comparable with the results of experimental studies in animals treated with psychoactive drugs (Kolb and Gibb, 2014, 2015) and in healthy human subjects after training (see references in May, 2011), which showed not only that plastic changes are region-specific, but also that different regions can express opposite changes. Moreover, the fact that the right FAT was anatomically intact can justify incremental changes on its microstructure in response to pharmacological and behavioral manipulation perhaps due to optimization of cholinergic activity, activity-dependent myelination, glial rearrangement, formation of new synapsis, or increased vasculature (May, 2011; Fields et al., 2017).

The relationship between improved speech fluency and decrement in the ASAF volume with both treatments is more difficult to interpret. While recent studies link the function of the ASAF with speech fluency (Fridriksson et al., 2013; Wang et al., 2013; Basilakos et al., 2014), the shrinkage of the right ASAF in JAM could, *a priori*, cast doubts on its participation in the recovery of speech fluency. Nevertheless, based on the evidence that different regions can express opposite plastic changes in humans (Kolb and Gibb, 2014, 2015), it is plausible that the intact right FAT may have taken over the function of the right dysfunctional ASAF. Moreover, a component of the lesion involved the right ASAF, so that the two interventions (drug and therapy) probably had less influence on repairing its structure. The causal role of the expansion of part of the area of tissue damage during the trial on inducing reductive changes in the ASAF is difficult to establish. An alternative explanation would be that cholinergic stimulation affected the microstructure of the right ASAF by reducing inflammation. The FAT and ASAF in the left hemisphere showed no relevant volume changes with either treatment.

### Repetition and the Arcuate Fasciculus

Repetition of words improved with both interventions (DP and DP-LLR therapy), whereas repetition of sentences from the PALPA battery was very difficult to JAM and it did not improve at all with either treatment. Repetition of clichès and novel phrases only improved with the combined treatment and performance on clichès declined thereafter, but gains in repetition of novel sentences remained highly significant in the first post-treatment evaluation. Thus, treatment with DP alone exerted a modest priming effect for novel sentence repetition only, yet the addition of behavioral training significantly boosted performance in both tasks. The superior improvement of novel sentences as compared to matched idiomatic clichès aligns well with findings from cases of CA after left hemisphere strokes treated with a similar strategy (Berthier et al., 2014). Although treatment with DP alone and combined DP-LLR therapy was associated with steady volume increments of the right DSAF, a segment of the AF implicated in verbal repetition (Saur et al., 2008), these changes were no longer apparent when this segment was compared to the one of healthy controls. As indicated in the previous section, it is possible that the right FAT mediated repetition, at least in part. Although the FAT seems to be unrelated to repetition performance in primary progressive aphasia (Catani et al., 2013), verbal repetition in healthy subjects increased the interaction of cortical areas (pre-SMA and dorsal premotor cortex) connected by the left FAT (Hartwigsen et al., 2013). VBM of cortical areas connected by the DSAF showed no changes with either intervention.

### Candidate Mechanisms for Structural Plasticity in White Matter Tracts

Comparisons of neuroimaging data at pre- and post-treatment phases in JAM revealed local events of plasticity in the right FAT and DSAF with no longitudinal plastic changes in the homologous tracts of the left hemisphere nor in the cerebral cortex connected by these tracts. Thus, it seems that cholinergic potentiation with DP alone primed selectively plastic changes in certain white matter tracts (FAT and DSAF) and the continued effect of this cholinergic agent acting in concert with repetitive LLR therapy harnessed activity-dependent plasticity of these white matter tracts. The causal relationship between the observed plastic changes and cholinergic modulation is elusive, but it concurs with the results of different lines of research (Mesulam et al., 1992, 2003; Raghanti et al., 2008; Bohnen et al., 2009; Hiraoka et al., 2009; Imamura et al., 2015). Postmortem analysis of the mesial frontal lobe (Raghanti et al., 2008), which is one of the anatomical origins of the FAT (Catani et al., 2013) and crucial for communicative intentions (Catani and Bambini, 2014), revealed dense clusters of cholinergic axons which probably represent local events of plasticity or circuitry rearrangement (Mesulam et al., 1992; Raghanti et al., 2008). An *in vivo* study using positron emission tomography (PET) and [11C]methyl-4-piperidinyl propionate acetylcholinesterase (AChE) in middle-aged and elderly non-demented subjects with periventricular white matter involvement of vascular origin was associated with reduced cortical cholinergic activity most likely due to interruption of ascending cholinergic projections in the white matter (Bohnen et al., 2009). In complementary terms, a histochemical study of a young patient with pure subcortical vascular lesions disclosed disruption of the ascending cholinergic pathways in the deep white matter, although some acetylcholine-rich fibers and cholinergic cortical neurons survived even in the areas of greatest cholinergic denervation (Mesulam et al., 2003). Moreover, a study in healthy subjects using PET and [5-(11)C-methoxy]-donepezil showed a moderate concentration of the radiotracer in some cortical areas (frontal and anterior cingulate gyrus) which are the origins of the FAT (Hiraoka et al., 2009; Catani et al., 2013). Finally, *in vitro* studies showed that treatment with DP, via stimulation of nicotinic receptors, rapidly increase oligodendrocyte differentiation and myelination (Imamura et al., 2015).

## Author Contributions

All authors listed, have made substantial, direct and intelectual contribution to the work, and approved it for publication. MB, ID-T, NR-V, IM-T, MT-P, DL-B, FA, and GD were involved in conception and design, acquisition of data, or analysis and interpretation of data. MB, ID-T, MT-P, IM-T, and GD performed language, cognitive and behavioral evaluations. MB, NR-V, JP-P, KT-H, FA, and DL-B interpreted neuroimaging data. MB, NR-V, JP-P, MT-P, DL-B, and GD drafted the article and revised it critically for important intelectual content.

## Conflict of Interest Statement

MB has received honoraria from Pfizer, Eisai, Janssen-España, Novartis, Lundbeck, and Nutricia and consultancy for fees from Merz, Eli Lilly, and GlaxoSmithKline. He has received speaking fees from Pfizer, Eisai, Janssen-España, Novartis, Lundbeck, and Nutricia. The other authors declare that the research was conducted in the absence of any commercial or financial relationships that could be construed as a potential conflict of interest.

## Footnotes

1 www.repiteconmigo.es

2 www.repiteconmigo.es

3 http://www.mrtrix.org/

4 http://nipy.org/nibabel/

5 http://www.fil.ion.ucl.ac.uk/spm/

6 http://www.trackvis.org/

7 http://www.fmrib.ox.ac.uk/fsl/

8 All statistical comparisons were performed using McNemar tests (two-tailed) unless specified.

9 Improvement in fluency of the WAB was significant when different parameters of connected speech were analyzed, but not when the methodology of the WAB to rate fluency in spontaneous speech was applied.
